# Economic Burden in Chinese Patients with Diabetes Mellitus Using Electronic Insurance Claims Data

**DOI:** 10.1371/journal.pone.0159297

**Published:** 2016-08-29

**Authors:** Yunyu Huang, Pepijn Vemer, Jingjing Zhu, Maarten J. Postma, Wen Chen

**Affiliations:** 1 Unit of PharmacoTherapy, -Epidemiology & -Economics, Department of Pharmacy, University of Groningen, Groningen, The Netherlands; 2 School of Public Health, Fudan University, Shanghai, China; 3 Institute of Science in Healthy Aging & healthcaRE (SHARE), University Medical Center Groningen (UMCG), University of Groningen, Groningen, The Netherlands; 4 Department of Epidemiology, University Medical Center Groningen (UMCG), University of Groningen, Groningen, The Netherlands; Institute of Endocrinology and Metabolism, ISLAMIC REPUBLIC OF IRAN

## Abstract

**Background:**

There is a paucity of studies that focus on the economic burden in daily care in China using electronic health data. The aim of this study is to describe the development of the economic burden of diabetic patients in a sample city in China from 2009 to 2011 using electronic data of patients’ claims records.

**Methods:**

This study is a retrospective, longitudinal study in an open cohort of Chinese patients with diabetes. The patient population consisted of people living in a provincial capital city in east China, covered by the provincial urban employee basic medical insurance (UEBMI). We included any patient who had at least one explicit diabetes diagnosis or received blood glucose lowering medication in at least one registered outpatient visit or hospitalization during a calendar year in the years 2009–2011. Cross-sectional descriptions of different types of costs, prevalence of diabetic complications and related diseases, medication use were performed for each year separately and differences between three years were compared using a chi-square test or the non-parametric Kruskal-Wallis H test.

**Results:**

Our results showed an increasing trend in total medical cost (from 2,383 to 2,780 USD, p = 0.032) and diabetes related cost (from 1,655 to 1,857 USD) for those diabetic patients during the study period. The diabetes related economic burden was significantly related to the prevalence of complications and related diseases (p<0.001). The overall medication cost during diabetes related visits also increased (from 1,335 to 1,383 USD, p = 0.021). But the use pattern and cost of diabetes-related medication did not show significant changes during the study period.

**Conclusion:**

The economic burden of diabetes increased significantly in urban China. It is important to improve the prevention and treatment of diabetes to contribute to the sustainability of the Chinese health-care system.

## Background

As a common chronic disease, diabetes is costly to health care systems in nearly all countries [1]. People with diabetes have more outpatient visits, use more medications, have a higher probability of being hospitalized, and are more likely to require emergency and long-term care than people without the disease [2]. In China, an estimated 20.8 million people had diabetes mellitus (DM) in 2000 [3] and this number increased markedly to 92.4 million in 2008 [4]. The updated data showed that the number of adult people with diabetes in China was 109.6 million in 2015 and this number is predicted to increase to 150.7 million by 2040 [5], approximately 11% of the Chinese adult population. This implies a considerable economic burden of diabetes and its complications making it an important public health challenge.

In China, electronic health information systems were developed at hospital and city level in urban areas, which could improve the quality of care and help to support the decision making. Access to valid electronic heath data is necessary for improvement of effectiveness evaluation, disease management and reimbursement policy-making. Unfortunately, a general lack of standardized, reliable and systematic coding of diagnoses and prescriptions in the Chinese electronic health information systems is common [6].

Optimal control of diabetes disease can mitigate costs of diabetes and complications. Although the Chinese Diabetes Society has published a guideline of prevention and treatment for Type 2 Diabetes Mellitus [7], the guideline is often not followed in real-world treatment and use of medication for diabetes [8]. This may lead to non-optimal control of the course of disease and reduce the affordability of treatment and care for diabetic patients. Existing domestic public health studies of diabetes in China mainly focus on prevalence and disease burden [9, 10]. Specific effectiveness studies and economic evaluations of diabetes related medications were also reported for Chinese patients [11–17]. However, data used in these studies were usually collected by household surveys or clinical trials and were usually based on single hospitals, which may lack data precision or external validity. There is a paucity of studies that focus on the economic burden in daily care using electronic health data.

The aim of this study is to describe the development of the economic burden of diabetic patients in a sample city in China from 2009 to 2011 using electronic data of patients’ claims records. The economic burden assessed includes diabetes complications and diabetes related diseases. As the potentially largest component of the costs, medication use and its related cost will be specifically detailed in the analyses.

## Methods

### Study design and cohort definition

This study is a retrospective, longitudinal study in an open cohort of Chinese patients with diabetes between 2009 and 2011.

The patient population consisted of all people living in a provincial capital city in east China (HZ), covered by the provincial urban employee basic medical insurance (UEBMI), one of the two nationwide urban basic medical insurances. This includes employees from provincial companies and governmental organizations in the sample city. We have chosen this sample city (HZ), as it is seen as a role model for other cities for their well-organized health information system [18], which ensures the quality of electronic data. The sample city is also a typical representative of eastern urban China considering the social economic development level. The records of all outpatient visits and hospitalizations in the electronic medical insurance claims database were tracked for inclusion.

We included any patient who had at least one explicit diabetes diagnosis or received blood glucose lowering medication in at least one registered outpatient visit or hospitalization during per calendar year in the years 2009–2011. Blood glucose lowering medications were identified by the strings of ‘A10A’ (insulins and analogues) and ‘A10B’ (blood glucose lowering drugs excluding insulins) contained in Anatomical Therapeutic Chemical (ATC) codes Patients who received blood glucose lowering medications but had diagnosis of polycystic ovary syndrome (PCOS) other than diabetes were excluded since evidence showed that some blood glucose lowering drugs were used for PCOS cases [19]. All diagnosis and prescription were set by doctors and recorded in the electronic database, which were trusted to reflect the valid and reliable information.

### Data extraction

Detailed claims records of outpatient visits and hospitalizations in each year were extracted for each included patient. Data were extracted in two types of datasets for each year. The first dataset contained the general information of each doctor visit, including patients’ internal ID (which can be tracked during different years), gender and age, visit type (outpatient visit or hospitalization), serial number of visit, date of visit, level of institution, length of stay, diagnosis and costs. Cost information contained total cost for the visit and the allocated costs, including 1) cost covered by the insurance scheme, 2) cost reimbursed by civil servant subsidies, and 3) cost covered by patients themselves, labeled out-of-pocket (OOP) payment. The second dataset contained detailed information for every treatment, examination, test, material and medications issued or prescribed during each visit, including name of issued items, form, unit price, quantity and reimbursement type in the insurance list. Dose information was not provided for any of the prescribed medication.

### Data standardization

In China, data management in electronic data systems generally lack standardization [20]. Although the Chinese name of diagnoses and medication names were recorded in the original datasets, those names could be various and without unified coding due to physician preference or a difference in structure of information system between hospitals. To standardize, we categorized diagnoses and recoded diabetes related medications based on their Chinese names for further analyses.

We categorized the diagnoses into 11 groups, which included four major complication groups, six diabetes related disease groups and a group for acute complications (S1 Table). This categorization was based on the Chinese guideline of prevention and treatment for diabetes[7]. The acute complications were identified by an experienced endocrinologist from one of the tertiary hospitals affiliated to Fudan University, Shanghai, China. All extracted diagnoses were screened for full name and terms indicating related diseases (e.g., “kidney” indicating nephropathy, “eye” indicating retinopathy) by the first and third author independently. Disagreements and indetermination were discussed with the endocrinologist to reach a consensus.

Both biomedicine (called Western medicine in China) and Chinese traditional medicine play important roles in patients’ health care in China. Biomedicine was recoded into ATC codes based on the Chinese names of medication in prescriptions. All unique biomedicine names in the datasets were recoded and double-checked by 12 medical students in two rounds.

All medication was categorized into six groups (S2 Table), based on the Chinese guideline of prevention and treatment for diabetes: insulins and analogues (ATC codes starting with ‘A10A’), all other blood glucose lowering drugs (ATC codes starting with ‘A10B’), antihypertensive medications (ATC codes starting with ‘C02’, ‘C03’, C07’, ‘C08’, ‘C09’) and lipid modifying medications (ATC codes starting with ‘C10’) within the biomedicine category, other biomedicine and Chinese traditional medicine. Due to the lack of official coding standards and systematic clinical evidence of effectiveness for diabetes, Chinese traditional medicines were analyzed as a single category.

### Data analysis

Cross-sectional descriptions of different types of costs, prevalence of diabetic complications and related diseases, medication use were performed for each year separately.

Different costs were defined as follows:

1. Total medical cost: total cost of all outpatient visits and hospitalizations recorded in the insurance database in one calendar year for included patients.
2. Diabetes related cost (DM cost): cost occurring in diabetes related medical visits (DM visits). Outpatient visits and hospitalizations were categorized as “diabetes related” when: (a) the diagnosis explicitly indicated diabetes or diabetic complications or related disease; OR (b) diabetes related biomedicines (as defined above) were prescribed during the visit.
3. Overall medication cost during DM visits: cost of all biomedicines and Chinese traditional medicines prescribed during the diabetes related visits.
4. Diabetes related biomedicine cost (DM biomedicine cost): cost of diabetes related biomedicines.

The reported costs in 2010 and 2011 were adjusted to 2009’s price using the Chinese official reported consumer price index (3.3% in 2010, 5.4% in 2011 [21]). All costs were reported in US dollars using the exchange rate of each year (1 US dollars = 6.831, 6.770 and 6.459 Chinese Yuan in 2009, 2010 and 2011, respectively [21]) to make the results more comparable to other published studies [1, 22, 23]. Differences of hospitalization rate, prevalence of complications and related diseases and DM biomedicine use between three years were compared using a chi-square test. Cost data and percentages were compared by the non-parametric Kruskal-Wallis H test. Differences between any two years for above-mentioned outcomes were compared by a post hoc pairwise comparison adjusted for multiplicity. The association of DM costs (log transferred due to skewed distribution) with number of complications and time was tested by multivariate linear regression with an interaction of these two factors. Data preparation and statistical analyses were performed using Stata SE Version 14.0 (Stata Corporation, College Station, TX).

### Ethics Statement

The data used in this study was anonymized before authors had access to the data. For research using anonymous electronic medical records no ethics committee approval is needed in China.

## Results

### Patient population

The studied cohort included 1,668, 1,797 and 2,078 patients from 2009 to 2011, respectively (Table 1). Almost all (97.8% of patients in 2009) were included in all three years. Approximately 65% patients were male. 80.9%, 79.6% and 76.2% patients were aged from 45 to 75 years old in 2009, 2010 and 2011, respectively. From 2009 to 2011, the number of diabetic patients gradually had an apparent increasing trend in the sample city.

**Table 1:** Demographic information of sampled patients.

		2009	2010	2011
Total patients		1,668	1,797	2,078
Gender	male	1,066(63.9%)	1,152(64.1%)	1,352(65.1%)
	female	602(36.1%)	645(35.9%)	726(34.9%)
Age group	0–30	7(0.4%)	7(0.4%)	10(0.5%)
	30–45	122(7.3%)	124(6.9%)	160(7.7%)
	45–60	638(38.3%)	675(37.6%)	772(37.2%)
	60–75	711(42.6%)	754(42.0%)	822(39.6%)
	75+	190(11.4%)	237(13.2%)	314(15.1%)

### Total annual medical cost

For all patients, the annual total cost per patient increased from 2,383 to 2,780 USD from 2009 to 2011 (p = 0.032), which was mainly caused by difference between 2009 and 2011 (adjusted p = 0.006) (Table 2). Annual DM cost averagely accounted for 70% of the total cost in 2009 and 2010 and increased from 1,655 to 1,857 USD in the study period, but decreased to 67.2% in 2011 (p = 0.001, adjusted p-value for 2009 vs 2011 = 0.047). Approximately 30% of the total costs were paid by patients themselves and the average percentage of this OOP payment decreased from 29.8% in 2009 to 27.7% in 2011 (p = 0.001, adjusted p-value for 2010 vs 2011 = 0.001, 2009 vs 2011 < 0.001).

**Table 2:** Average annual costs per patient (USD).

	2009	2010	2011	p-value of 3-year comparison	Multiplicity adjusted p values		
					09vs10	10vs11	09vs11
**Annual costs per patient**							
Number of patients	1,668	1,797	2,078				
Total medical cost	2,382.5	2,556.2	2,780.1	0.032	0.424	0.313	0.006
OOP payment (% of total medical cost)	781.7(29.8%)	807.8(29.1%)	822.3(27.7%)	0.001*	1.000	0.331	0.047
DM cost (% of total medical cost)	1,655.0(70.3%)	1,719.4(70.0%)	1,856.5(67.2%)	<0.001*	1.000	0.001	<0.001
**Annual costs per patient of patients with hospitalizations**							
Number of patients (% of total patients)	287(17.2%)	361(20.1%)	420(20.2%)	0.038	0.030	0.924	0.019
Total medical cost	6,301.4	6,249.9	7,093.9	0.558	1.000	0.456	0.412
OOP payment (% of total medical cost)	1,972.5(32.2%)	1,989.1(33.3%)	2,032.8(31.8%)	0.820*	1.000	0.798	1.000
DM cost (% of total medical cost)	4,768.2(75.9%)	4,480.8(74.3%)	5,295.9(72.0%)	0.073*	1.000	0.437	0.058
Hospitalization cost (% of total medical cost)	3,791.4(52.5%)	3,514.1(51.2%)	4,626.3(53.2%)	0.596*	1.000	0.701	1.000
**Annual costs per patient of patients without hospitalizations**							
Number of patients (% of total patients)	1,381(82.8%)	1,436(79.9%)	1,658(79.8%)	0.038	0.030	0.924	0.019
Total medical cost	1,568.0	1,627.7	1,687.3	0.221	0.543	0.940	0.052
OOP payment (% of total medical cost)	534.2(29.3%)	510.8(28.0%)	515.6(26.6%)	<0.001*	0.745	0.545	0.036
DM cost (% of total medical cost)	1008.0(69.1%)	1,025.2(69.0%)	985.3(66.0%)	0.001*	1.000	0.002	0.001

* Comparison of percentages of total medical cost

The proportion of patients with hospitalizations increased from 17.2% to 20.2% (p = 0.038, adjusted p-value for 2009 vs 2010 = 0.030, 2009 vs 2011 = 0.019) during the study period. For patients needing hospitalization, the economic burden was much heavier. The average annual cost per patient with at least one hospitalization in a year (6,301 USD in 2009) was more than four-fold the costs per patient with only outpatient visits (1,568 USD in 2009). Annual hospitalization cost increased from 3,791 in 2009 to 4,626 USD in 2011, but this increase was not significant (p = 0.596). The hospitalization cost was the major drive for the annual costs, which accounted for more than 50% of annual total cost. Patients needing hospitalization had a higher percentage of OOP payment and DM cost comparing to their counterparts without hospitalizations. In fact, these two percentages decreased from 2009 to 2011 in patients having no hospitalizations (p<0.001 and 0.001, respectively), but not in patients needing hospitalization.

### Prevalence of diabetic complications and related diseases

Based on recorded diagnoses, 60.9% of DM patients had at least one diabetic complication or related disease in 2009 and this percentage increased to 71.2% in 2011 (p<0.001) (Table 3). The percentage of patients with only one complication or related disease decreased from 22.2% to 21.5% from 2009 to 2011. Percentages of patients with two or more complications and related diseases all increased. Hypertension was the related disease of highest prevalence in the included patients, followed by cardio- and cerebral-vascular diseases and hyperlipidemia.

**Table 3:** Prevalence of diabetic complications and related diseases.

	Number of DM patients (% of total patients)*		
	2009	2010	2011
Total patients	1,668	1,797	2,076
Patients with complications	1015(60.9%)	1159(64.5%)	1479(71.2%)**
With one complication	371(22.2%)	404(22.5%)	447(21.5%)
Two complications	274(16.4%)	333(18.5%)	442(21.3%)
Three complications	189(11.3%)	222(12.4%)	315(15.2%)
Four or more complications	181(10.9%)	200(11.1%)	275(13.2%)
**Prevalence of different diseases**			
Hypertension	733(43.9%)	853(47.5%)	1,082(52.1%)
Cardio- and cerebral vascular diseases	429(25.7%)	539(30.0%)	701(33.7%)
Hyperlipidemia	294(17.6%)	314(17.5%)	422(20.3%)
Neuropathy	275(16.5%)	288(16.0%)	412(19.8%)
Retinopathy	236(14.2%)	297(16.5%)	382(18.4%)
Nephropathy	146(8.8%)	157(8.7%)	242(11.7%)
Acute complications	96(5.8%)	99(5.5%)	122(5.9%)
Fatty liver	82(4.9%)	66(3.7%)	105(5.1%)
Diabetic foot	6(0.4%)	7(0.4%)	31(1.5%)
Hyperuricemia	10(0.6%)	13(0.7%)	22(1.1%)
Troisier-Hanot-Chauffard syndrome	-	1(0.1%))	5(0.2%)

* Since patients can have more than one complications, the proportions didn’t add up to 100%; sorted on % in 2011

** Chi-square test of three year comparison of number of patients with complications: p<0.001

### Diabetes related costs of patients with complications and related diseases

**Fig 1:**
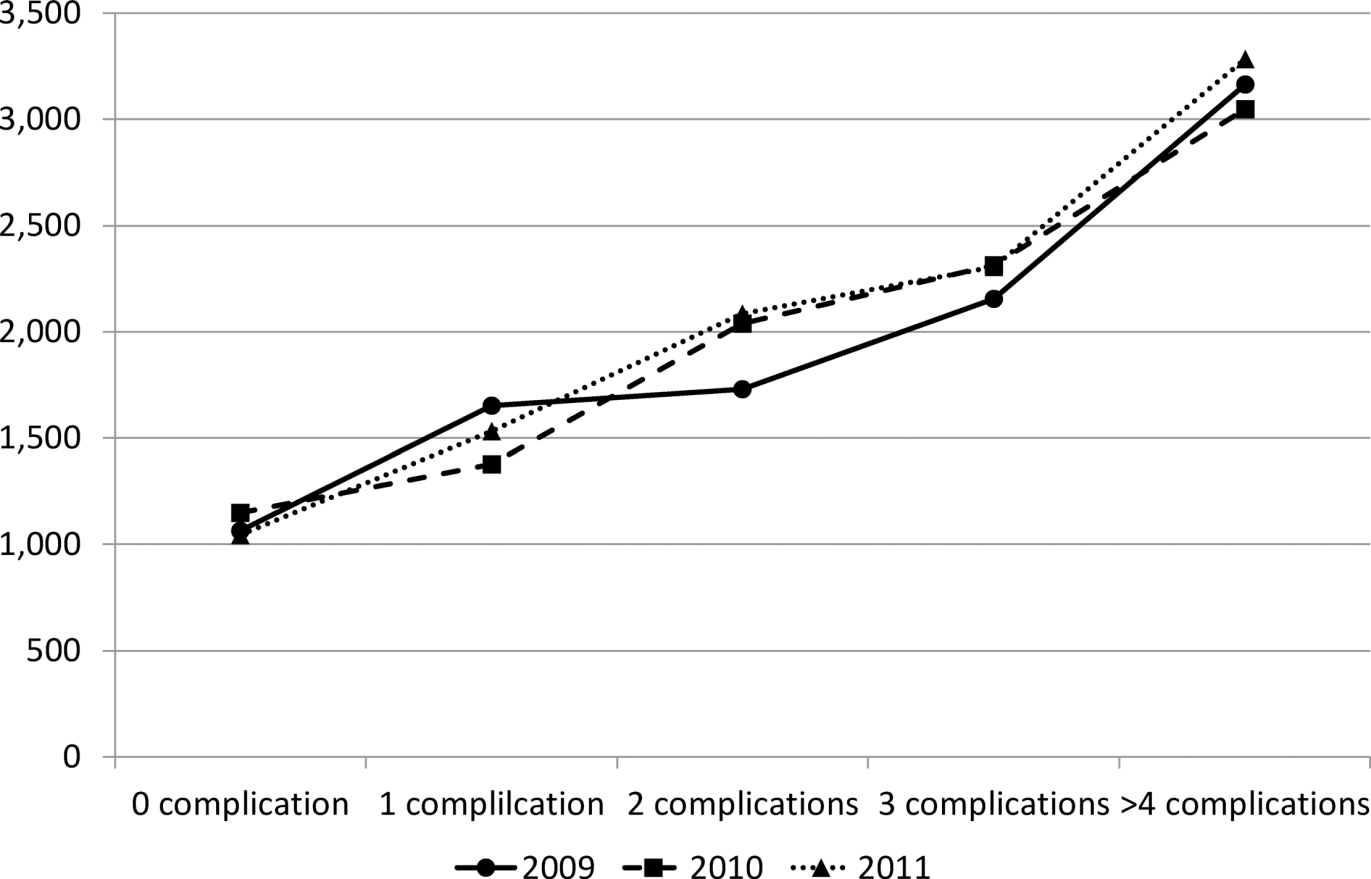
Diabetes related costs for patients with complications and related diseases (USD).

The number of complications and related diseases was significantly related to the direct medical burden of diabetes treatment, with more diseases causing higher costs (p<0.001 in all groups with one or more complications comparing to the reference group with no complications) (Fig 1). The average annual DM cost didn’t show a significant growing trend in 2010 comparing to 2009 as reference (p = 0.383), but increased significantly from 2009 to 2011 (p = 0.035). There was no interaction effect between number of complications and time.

### Diabetes related medication use and costs

Overall medication cost during DM visits per patient increased from 1,335 to 1,383 USD in the studied years (p = 0.021, adjusted p-value for 2009 vs 2011 = 0.039) (Table 4). This increase was mainly due to increased cost for biomedicines (p = 0.012). The vast majority of DM costs was accounted by medication cost (90%), although this percentage slowly decreased overtime (p<0.001, adjusted p-value for 2010 vs 2011 = 0.001, 2009 vs 2011 < 0.001).

**Table 4:** Diabetes related medication use and costs (USD).

	2009	2010	2011	p-value of 3-year comparison	Multiplicity adjusted p values		
					09vs10	10vs11	09vs11
Annual overall medication cost during DM visits per patient	1,335.4	1,367.7	1,383.4	0.021	1.000	1.000	0.719
%of DM cost	91.2%	90.1%	88.2%	<0.001*	0.147	0.001	<0.001
Annual biomedicine cost per patient	1,144.3	1,188.1	1,185.3	0.012	0.856	1.000	0.732
Annual traditional medicine cost per patient	222.5	215.5	239.9	0.685	1.000	0.367	0.476
Annual DM biomedicine cost	736.5	746.9	698.9	0.284	0.401	0.057	1.000
%of overall medication cost during DM visits	68.7%	68.7%	69.3%	0.750*	1.000	1.000	1.000
**Blood glucose lowering biomedicines**							
Number of users (% of total patients)	1,638(98.2%)	1,763(98.1%)	2,040(98.2%)	0.978	0.838	0.884	0.945
Annual cost per patient (% of overall medication cost during DM visits)	476.8 (47.7%)	481.3 (47.7%)	437.1 (46.5%)	0.295*	1.000	0.642	0.608
**Antihypertensive biomedicines**							
Number of users (% of total patients)	1,164(69.8%)	1,290(71.8%)	1,497(72.0%)	0.268	0.195	0.861	0.130
Number of RAAS inhibitor users (% of total patients)	872(52.3%)	957(53.3%)	1,083(52.1%)	0.754	0.565	0.479	0.922
Annual cost per patient (% of overall medication cost during DM visits)	306.8 (25.4%)	301.5 (24.9%)	285.0 (25.9%)	0.467*	1.000	0.493	1.000
**Lipid-modifying biomedicines**							
Number of users (% of total patients)	527(31.6%)	588(32.7%)	751(36.1%)	0.008	0.478	0.026	0.004
Annual cost per patient (% of overall medication cost during DM visits)	174.5 (12.4%)	182.4 (12.2%)	178.4 (13.1%)	0.318*	1.000	0.559	0.874

* Comparison of percentages

DM biomedicine cost didn’t show a significant trend (p = 0.284). The cost of blood glucose lowering biomedicines, including insulins and analogues and all other blood glucose lowering biomedicines, accounted for a major part (47% on average) of overall medication cost during DM visits. Approximately 70% of all patients used antihypertensive medicines, with the costs accounting for 25% of overall medication costs during DM visits. The percentage of hypertensive biomedicine users and the proportion of these medication cost in the overall medication cost during DM visits remained stable from 2009 to 2011. The percentage of patients using lipid-modifying biomedicines was much lower in 2009 (31.6%), but increased to 36.1% in 2011.

## Discussion

To our knowledge, this is the first study analyzing the direct economic burden in diabetic patients using longitudinal data from electronic medical insurance claims database. Our results showed an increasing trend in total medical cost and diabetes related cost for diabetic patients during the study period. The diabetes related economic burden was significantly related to hospitalization and the prevalence of complications and related diseases. The overall medication cost during diabetes related visits also increased from 2009 to 2011. But the use pattern and cost of all three groups of DM biomedicine didn’t show significant changes during the study period.

For Chinese patients, studies on economic burden of diabetes from the patient perspective emerged after 2000 [10]. Those studies usually adopted a cross-sectional design and data were collected in the hospitals or recalled by patient interviews [23–28]. Although the expansion of the number of studies in different cities enhanced the crosswise comparison, existing studies didn’t conduct historical comparison usually due to the cost of data collection. In this study, economic burden could be compared over three years in basically the same population (97.8% of patients in the first year could be followed for three years) using electronic health records. Because the UEBMI scheme was highly developed in the sample city (HZ) and covered a stable population, the increase of included patients from 2009 to 2011 indicates a rapid growth of diabetes prevalence in urban China. Considering that our inclusion criteria excluded those patients who had no registered diagnosis or records of hypoglycemic drug use but may have diabetes, there actually may be more diabetic patients in this insured population. The age distribution of studied population was in accordance with the conclusions of diabetes prevalence in the existing literature [22]. The gap of patient numbers between male and female was mainly due to the gender imbalance in the employers working in provincial companies and organizations.

Although the prevalence of different diabetic complications and related disease haven’t been systematically investigated nationwide in China, the Chinese guideline of prevention and treatment for diabetes summarized the epidemiological situation of those diseases using evidence from individual studies [7]. Compared to those data, our sample showed a mildly higher prevalence of hypertension (52.1% in 2011), similar prevalence of cardio- and cerebral-vascular disease (33.7% in 2011) and lower prevalence of nephropathy, retinopathy, neuropathy and diabetic foot. The differences may be partly because of our strict diagnosis categorization based on screening of diagnosis name and terms for those diseases with varied pathogeneses and symptoms. Especially for neuropathy, studies found that 60%-90% of diabetic patients may have this complication but 30%-40% of them have no symptoms and thus are undiagnosed [7]. Both those undiagnosed patients and limitation of our diagnosis categorization may underestimate the prevalence.

Data from 2013 estimated that the worldwide average annual cost of treating and managing diabetes was 1,437 USD per person [5]. Results in this study showed that the average annual DM cost per patient increased from 1,655 to 1,857 USD. This trend reflected that diabetes caused increasing economic burden in the medium and large-sized cities in China. One study concluded that the average annual growth rate of the direct medical cost of DM was 19.90% during 1993 to 2003 in China, which was much larger than the average annual growth rate of GDP (12.77%) [29]. The average annual growth rate of DM cost in our studied population (5.9% from 2009 to 2011) seemed not to exceed the rapid growth of GDP per capita in the sample city (15.33% from 2009 to 2011 [30]). But when comparing to the disposable income per capita in the sample city (3,937, 4,290 and 4,792 USD in 2009, 2010 and 2011, respectively [30]), the OOP medical cost can still be considered a heavy burden for the diabetic patients (approximately 20% of disposable income).

Our results confirm the decisive role of hospitalization in direct medical costs reported before [10, 22, 31]. The results also showed an increase in percentage of patients needing hospitalization. The high rates of hospitalization underline the poor condition of prevention for diabetes in China and contribute to the increase of total medical cost. Patients needing hospitalization usually had more serious conditions (higher percentage of total medical cost for DM cost) and faced higher OOP payment burden. Comparing to the disposable income per capita, OOP cost for patients needing hospitalization accounted for over 40% of the disposable income, a great problem for the individual. Our study showed that DM cost increased with the number of complications. This result underlines the evidence from other studies showing that complication-induced direct medical cost causes a high economic burden in diabetic patients [22, 27]. The longitudinal data showed a significant growth of percentage of patient with at least one complication. The linear relationship was statistically significant between the number of complications and DM cost.

Medications were proved to be the main form of treatment in terms of costs for controlling blood glucose and other diabetes complications and related diseases [32]. Our results also showed an increase of overall medication cost occurring during DM visits. But medication cost percentage of total DM cost decreased. This may be explained by the promotion of a comprehensive medicine policy after launch of the Chinese new health reform in 2009, which regulated physicians’ prescription behavior to provide inexpensive generic DM biomedicines to improve the medication use. Meanwhile, cost of diabetes related biomedicines didn’t show a growing trend. Therefore, the increasing of total medical cost, DM cost and overall medication cost may be explained by other reasons. More serious course of disease, more hospitalizations, more drugs and treatments for concomitant diseases may all contribute to cause the increased costs.

The International Diabetes Federation Clinical Guidelines Task Force’s global guideline for T2DM calls for the use of a statin and a renin-angiotensin-aldosterone system (RAAS) inhibitor in all persons with T2DM, regardless of lipid and blood pressure levels [33]. Our results showed a low number of these medications users, although the percentage of lipid-modifying medication users increased significantly from 2009 to 2011. China still needs to improve the prevention and treatment pathway for diabetes and thus prevent the negative impacts induced by growth of future hospitalizations, disability, mortality, and medical care costs.

Our study used the electronic medical data instead of reported or surveyed data. Because insurance identity cards are mandatorily used when patients see a doctor, we believe these electronic data to be accurate and credible. These data also contained information on medical visits occurring in all levels of health institutions, which was the main limitation in individual hospital based studies. Despite this strength, this study still had limitations. First, the definition of diabetes patients in our study excluded those patients who had no DM visits registered in the electronic database but may have diabetes. In addition, our data didn’t differentiate type 1 and type 2 diabetes, although the prevalence of type 1 diabetes in China was estimated as lowest around the world [34]. Second, our definition of DM cost included all costs during diabetes and complications based on the diagnosis name. Although other diseases irrelevant to diabetes, e.g. trauma, were excluded, we assumed those complications that could in principal have other causes than diabetes was diabetes related. For example, a patient with a hospitalization for nephropathy, which could be caused by high blood pressure alone, was assumed to be caused by diabetes, since all patients in our study were defined as having diabetes. This is an overestimation, but we expected this overestimation to be very small. Third, we believe that the information for the electronic data is trustworthy based on the well-structured information system, but there could be possibilities of mistakes in the raw data level. Furthermore, the procedure of recoding prescriptions and diagnoses may introduce misclassification of medications and diagnoses. Fourth, our data didn’t contain the population other than provincial UEBMI beneficiaries. Compared to the other basic medical insurance scheme in urban areas, urban residents’ basic medical insurance (URBMI), UEBMI has higher reimbursement level for both outpatient and inpatient visits. In addition, URBMI covers unemployed adults and elderly people with poorer economic status. Both higher reimbursement level and better economic status for UEBMI beneficiaries would encourage them to use more health services and spend more money on health. Thus the results may estimate higher economic burden comparing to burden for patients with other insurance scheme. Lastly, the indirect costs in diabetes have important societal influence. However, our electronic data is not suitable to calculate indirect costs. The impact of indirect costs will be an important topic for future research.

## Conclusion

The economic burden of diabetes increased significantly in urban China, with a significant OOP burden for patients needing hospitalization. As diabetes prevalence is expected to grow in the future, economic burden of diabetes will continue to weigh heavily on health budgets. For the relatively new system of universal health coverage in China, the increasing economic burden of diabetes will aggravate the contradiction between limited health resources available and increasing health care demand. Therefore, it is important to improve the prevention and treatment of diabetes to contribute to the sustainability of the Chinese health-care system.

## Supporting Information

S1 Table Categories of diagnoses for data standardization. (DOCX)

S2 Table Diabetes related biomedicines included in the data analyses. (DOCX)
