# Supplementary material for: Economic Burden in Chinese Patients with Diabetes Mellitus Using Electronic Insurance Claims Data
Source: PLoS One. 2016 Aug 29;11(8):e0159297. doi: 10.1371/journal.pone.0159297 (PMC5003380; doi:10.1371/journal.pone.0159297)
Supplement: S1 Table — (DOCX) [file pone.0159297.s001.docx]

**S1 Table. Categories of diagnoses for data standardization**

| **Disease categories** | **Keywords contained in the diagnosis names** |
| --- | --- |
| Chronic diabetes complications |  |
| Nephropathy | Nephro- (e.g. nephropathy, nephritis); kidney (e.g. kidney transplantation); renal (e.g. renal failure); urine (e.g. uremia, proteinuria) |
| Retinopathy | Retino- (e.g. retinopathy, retinal vasculopathy); fundus; eye; cataract; maculopathy; vitreous; vision |
| Neuropathy | Neuro- (e.g. neuropathy, neuritis, neurosis); nerve; radiculopathy; frequent micturition; insomnia; night sweat; sleep disorders |
| Diabetic foot | Foot; lower extremities |
| Diabetes related diseases |  |
| Carodio- and cerebral vascular disease | Vascular; cardio-; cerebro-; heart; stroke; atherosclerosis; coronary |
| Hypertension | hypertension |
| Hyperlipidemia | Hyperlipidemia; hypercholesteraemia; dyslipidemia |
| Fatty liver | Fatty liver |
| Hyperuricemia | Hyperuricemia |
| Troisier-Hanot-Chauffard syndrome | Troisier-Hanot-Chauffard; diabetic pigmentary cirrhosis |
| Acute diabetes complications | Ketoacidosis; nonketotic hyperosmolar coma; hypoglycemic coma; infection |
