# Supplementary material for: Economic Burden in Chinese Patients with Diabetes Mellitus Using Electronic Insurance Claims Data
Source: PLoS One. 2016 Aug 29;11(8):e0159297. doi: 10.1371/journal.pone.0159297 (PMC5003380; doi:10.1371/journal.pone.0159297)
Supplement: S2 Table — (DOCX) [file pone.0159297.s002.docx]

**S2 Table. Diabetes related biomedicines included in the data analyses**

| **Medication categories** | **Medication names** |
| --- | --- |
| Blood glucose lowering medications, excluding insulins  (ATC codes start with ‘A10B’) | metformin; glibenclamide; glipizide; gliclazide; gliquidone; glimepiride; acarbose; voglibose; rosiglitazone; pioglitazone; sitagliptin; repaglinide; nateglinide; exenatide; miglitol; mitiglinide; saxagliptin; vildagliptin; linagliptin; alogliptin; liraglutide |
| Insulins and analogues  (ATC codes start with ‘A10A’) | Insulins and analogues for injection, fast-acting (insulin, insulin aspart, insulin lispro, insulin glulisine);  intermediate-acting;  long-acting (insulin, insulin glargine, insulin detemir);  intermediate- or long-acting combined with fast-acting (insulin, insulin aspart, insulin lispro) |
| Antihypertensive medications  (ATC codes start with ‘C02’, ‘C03’, C07’, ‘C08’, ‘C09’) | ‘C02’ Antihypertensives: doxazosin; prazosin; urapidil; terazosin  ‘C03’ Diuretics: furosemide; hydrochlorothiazide; indapamide; amiloride; triamterene; spironolactone  ‘C07’ Beta blockers: metoprolol; bisoprolol; atenolol; propranolol; betaxolol; labetalol; carvedilol; arotinolol  ‘C08’ Calcium channel blockers: nifedipine; amlodipine; felodipine; lacidipine; nicardipine; nitrendipine; lercanidipine; verapamil  ‘C09’ Renin–angiotensin–aldosterone system inhibitors: captopril; benazepril; enalapril; cilazapril; fosinopril; perindopril; ramipril; Lisinopril; imidapril; losartan; valsartan; irbesartan; candesartan; telmisartan; olmesartan |
| Lipid modifying medications  (ATC codes start with ‘C10’) | Fenofibrate; gemfibrozil; bezafibrate; lovastatin; simvastatin; pravastatin; fluvastatin; atorvastatin; rosuvastatin; pitavastatin; nicotinic acid; colestyramine; colestipol; ezetimibe; policosanol |
| Other Biomedicine | Any biomedicine not mentioned above |
| Chinese traditional medicine | Any medication not included in biomedicine. |
